# Patient Attitudes Toward Blood‐Based Biomarker Testing for Alzheimer's Disease in Primary Care: Anticipated Results from the BLOOM‐AD Study

**DOI:** 10.1002/alz70860_103795

**Published:** 2025-12-23

**Authors:** Selina Quinones, Joseph M Curran, Roscoe Bell, Jeff Williamson, Goldie S Byrd, Laura D Baker, Suzanne Craft, Andrea M Russell, Rebecca Lovett, Stephanie Batio, Jeimmy Hurtado, Morgan Bonham, Wei Huang, Adam Brickman, Michael S Wolf, Michelle M Mielke, Chinedu T Udeh‐Momoh

**Affiliations:** ^1^ Wake Forest University School of Medicine, Winston‐Salem, NC, USA; ^2^ Division of Public Health Sciences, Wake Forest University, School of Medicine, Winston‐Salem, NC, USA; ^3^ Northwestern University, Chicago, IL, USA; ^4^ Department of Neurology, Columbia University, New York, NY, USA; ^5^ Brain and Mind Institute, Aga Khan University, Nairobi Province, Nairobi, Kenya

## Abstract

**Background:**

Blood‐based biomarkers (BBMs) provide a less invasive and more accessible alternative to CSF or PET to diagnosis Alzheimer's disease (AD) among individuals with cognitive impairment. However, limited research exists on patient perspectives toward BBMs. BLOOM‐AD aims to explore patient attitudes toward BBMs, with a focus on identifying concerns, preferences, and disparities across diverse populations.

**Methods:**

The BLOOM‐AD study will recruit 1500 adults aged 50+ from Atrium Health primary care clinics. Participants will complete a 15‐minute survey assessing demographics, clinical health status, personal and family history of memory loss, as well as knowledge and attitudes toward AD and AD‐BBMs. Statistical testing will be done across the demographic factors of race/ethnicity, socio‐demographics, health status and sex.

**Results:**

We will have enrolled 1000 patients into the study by April 2025, and the full complement by summer 2025 when data collection will be completed. It is anticipated that this survey will precipitate a high interest in BBMs, particularly if biomarker results inform care planning, testing is covered by insurance, and PCPs endorse its use. However, we expect differences across racial and ethnic lines, with historically marginalized communities showing greater skepticism towards BBMs due to past healthcare inequities. Barriers related to health literacy and mistrust of healthcare services are expected. These findings may identify differences in BBM acceptance and barriers among diverse populations, who historically face greater health disparities in AD diagnosis and care. The results will guide future approaches for equitable, patient‐centered implementation of BBMs in primary care.

**Conclusions:**

The BLOOM‐AD study will deepen our understanding of patient perspectives on BBMs in primary care, with a focus on diverse populations. These insights will inform strategies to improve outreach, address disparities, and promote early AD detection and prevention, ensuring equitable access and addressing patient concerns in clinical practice.

